# Advancing standards in biomedical image analysis validation: A perspective on Metrics Reloaded

**DOI:** 10.1002/ctm2.70237

**Published:** 2025-08-31

**Authors:** Annika Reinke, Minu D Tizabi, Lena Maier‐Hein

**Affiliations:** ^1^ Division of Intelligent Medical Systems German Cancer Research Center (DKFZ) Heidelberg Heidelberg Germany; ^2^ German Cancer Research Center (DKFZ) Heidelberg, Helmholtz Imaging Heidelberg Germany; ^3^ National Center for Tumor Diseases (NCT), NCT Heidelberg, a partnership between DKFZ and University Medical Center Heidelberg Heidelberg Germany; ^4^ Faculty of Mathematics and Computer Science, Heidelberg University Heidelberg Germany

Over the past years, medical artificial intelligence (AI) research has seen tremendous progress and found entrance into various disciplines of clinical medicine, such as medical imaging. However, a—frequently unspoken—part of the truth is that the majority of newly developed AI methods do not see the light of translation into clinical practice.^1^ This lack can, to a large extent, be attributed to flaws in the robust and clinically useful validation of AI methods: in the absence of meaningful performance validation that takes into account the specific properties of the underlying clinical task, progress cannot be measured and clinical usability of a new method cannot be gauged. In other words, we spend vast resources to develop new algorithms, yet cannot trust them to perform well under real‐world clinical conditions, for example, marked by the presence of data shifts (see, e.g., Castro et al.^2^), poor data quality (see, e.g., Jogan et al.^3^), or different scanners (see, e.g., Kilim et al.^4^). Despite its tremendous importance, validation is often still treated as algorithm development's poor cousin: neither as glamorous nor as financially attractive as the latter, it is plagued by poor scientific practices and, to date, a lack of standards.

This becomes particularly evident in the case of validation metrics: In our recent sister publications,^5^, ^6^ we showed that choosing inadequate performance metrics (for example, by popularity) that do not reflect the clinical needs is both a prevalent common practice and a dangerous one: In the segmentation of brain magnetic resonance imaging (MRI) images for tumour detection, for instance, an AI algorithm considered as the state of the art could achieve impressive scores of a popular validation metric, yet consistently fail to detect small, clinically significant tumour lesions—with potentially fatal consequences for patients.

The choice of metrics in medical imaging is seemingly endless—and so are the pitfalls into which we can stumble when selecting and applying them. How, then, to identify metrics that we can trust to properly reflect an algorithm's real‐world clinical usability? This is where *Metrics Reloaded* comes in: *Metrics Reloaded* is the first comprehensive recommendation framework that guides researchers in the problem‐aware selection of clinically meaningful performance metrics in medical imaging (see Figure 1). It supports recommendations for any image‐based task related to the classification of the image (e.g., based on a computed tomography (CT) image, decide if a tumour is malignant or benign), object detection (e.g., tumour detection and localization in a CT image), or pixel and/or object segmentation level (e.g., outlining a tumour in a CT image). It thus follows a task‐agnostic approach, which could be achieved by abstracting from the actual underlying medical problem to a set of specific properties that affect metric choice. We abstract by asking questions like, “Are structure boundaries of specific interest or is it sufficient to focus on the object center?”, “Are there small structure sizes within the image?”, “Are the different classes imbalanced?”, etc.

**FIGURE 1:**
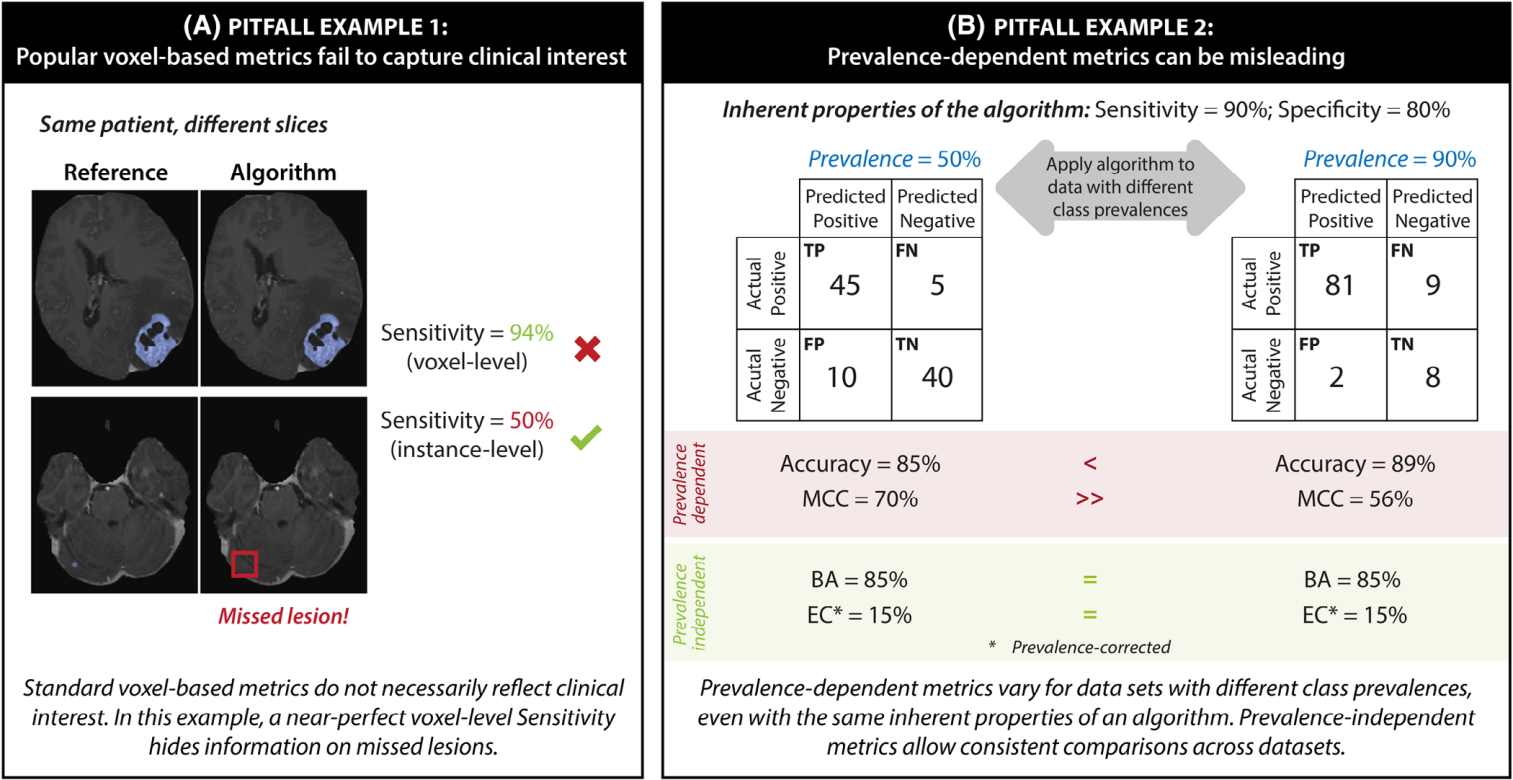
Examples of metric pitfalls. (A) In this example, voxel‐based SSensitivity does not adequately assess the tumour detection capabilities of an algorithm as it does not capture the algorithm's failure to detect small (but clinically highly relevant) lesions. The use of instance‐level sensitivity reveals poor performance. (B) The performance of an algorithm with fixed properties (e.g., Sensitivity of 0.9 and Specificity of 0.8) can appear vastly different when assessed on datasets with different prevalence and using prevalence‐dependent metrics (here: Accuracy and Matthews Correlation Coefficient (MCC)(). In contrast, prevalence‐independent metrics (here: Balanced Accuracy (BA) and Expected Cost EC)) enable consistent validation and comparison across datasets, regardless of prevalence. Figures were adapted from Reinke et al.^6^ ^.^

Based on answering those questions, our framework generates a “Problem Fingerprint” which then allows for the selection of appropriate metrics for each problem. The metrics recommended for a problem involving the accurate delineation of structure boundaries, for instance, would thus differ from those for a problem only requiring a rough localization of the structure centre—as they should.

To ensure the comprehensiveness of the problem fingerprints and the pitfalls commonly encountered in current validation practice, *Metrics Reloaded* was developed by a highly diverse, multidisciplinary expert consortium, comprising more than 70 international thought leaders with various expertise (e.g., machine learning—various domains, clinicians, biologists, statisticians, epidemiologists, …). Consensus was reached by a Delphi process. We see this as a potential blueprint for further standardization efforts in AI algorithm validation across different domains.

One highly generalizable aspect of *Metrics Reloaded* is its recommendation to not use a single metric for performance assessment. By definition, each metric comes with specific, task‐dependent pitfalls. An overlap‐based metric, for example, which measures the overlap between a reference and a predicted segmentation, is not able to capture the object shape properly. On the other hand, a boundary‐based metric compares the outline of reference and prediction, however, may miss holes inside an object. Both metrics combined would complement each other and give a full picture of the algorithm's performance.

To broaden the applicability of *Metrics Reloaded*, we included community feedback. As a result, the framework features shortcuts for 21 common use cases, so no need to run the whole process if one of those use cases corresponds to yours!

Of note, while technical researchers are typically interested in technical, mathematically inspired metrics, clinicians often prefer simpler variants. So, for a clinician, rather than calculating how many boundary pixels were correctly assigned by an algorithm for liver segmentation, a simple metric such as absolute liver volume would suffice. Therefore, we included the possibility of adding application‐specific metrics to the metric pool which reflect the final medical use case. We also encourage researchers to use non‐reference‐based metrics in addition to the ones suggested, that is, metrics that do not compare an expert reference to a prediction. This could include metrics measuring a method's runtime, computational complexity, or carbon footprint.

By nature, the original *Metrics Reloaded* publications are very long and harder to read. To facilitate adoption by the scientific community, we thus released an online toolkit^1^, which guides researchers through the entire process of metric selection while making them aware of the pitfalls to be sidestepped. It also includes the first comprehensive educational resource on validation in medical imaging—providing researchers with a common access point to previously widely scattered and/or outright inaccessible information. Notably, this includes an illustrated list of metric‐related pitfalls and a cheat sheet for each metric with relevant information, such as its formula, description, relevant relations, multi‐class definition, prevalence dependency, further limitations, associated pitfalls, and more. Being educated about metric relations is particularly important in avoiding confusion when clinicians interact with technical medical imaging researchers, or vice versa: In different disciplines, the same metric may be known by different names. Prominent examples include Recall and Sensitivity or F1 Score and Dice Similarity Coefficient (DSC) and have indeed made publications before: Based on an analysis by Reinke,^7^ three challenges in the period of 2018 to 2022 based their final rankings on two identical metrics. Other metrics (such as DSC and the Intersection over Union (IoU) or Accuracy and Error Rate) are very similar (mathematically related) and should not be reported together as they add no information and could mislead users into overestimating an algorithm's capabilities.

Furthermore, *Metrics Reloaded* provides standard metric implementations as part of the Medical Open Network for AI (MONAI) framework^2^. These are especially handy when it comes to the next step after metric selection: metric application. Not all metrics are straightforward to implement and based on chosen libraries, scores may differ. For example, the procedure of extracting an object's boundary is not standardized and may yield very different results.^6^ Once again, the underlying clinical needs must be reflected.

The field of medical imaging algorithm validation is wide, and its pitfalls are manifold. To conclude that the observed flaws are solely the fault of individual researchers would, however, be inappropriate: common practice in a research field largely comes down to education and incentivization. In both, the broader situation can be seen at fault: focus and incentives are provided for the development of ever‐new models; students are encouraged to develop their own model but are barely trained in validation, or the treacheries of unreflected metric use. Often, training only encompasses a few easy‐to‐understand metrics (e.g., Accuracy) which, unsurprisingly, fail to reflect the complexity of many real‐world scenarios. It is understandable that researchers tend to use metrics they know or try to identify suitable metrics by checking what other researchers have used in their publications (for example, we found that a quarter of biomedical image analysis competitions between 2018 and 2022 justified their metric choice by commonality, while more than half of competitions did not provide any justification at all^7^). In this manner, poor validation practices can easily propagate. *Metrics Reloaded* aims to disrupt this vicious cycle by both raising awareness and educating researchers as well as facilitating good scientific practice. Its presence at summer schools, seminars and large professional societies in the field has already ensured a wide reach.

So, what is next? Building upon its success, we are currently expanding *Metrics Reloaded* to other medical imaging problems, such as image reconstruction or synthesis, as well as to other domains, notably surgery. However, medical imaging algorithm validation extends way beyond metrics, encompassing as yet uncharted areas such as data selection. We strongly encourage research in this area, literally “re‐searching” common practice—as we have seen, treasures may await those who venture out into the unglamorous depths of algorithm validation.

## Footnotes

1
https://metrics‐reloaded.dkfz.de/

2
https://github.com/Project‐MONAI/MetricsReloaded/tree/main
